# Transcriptomic Characterization of the South American Freshwater Stingray *Potamotrygon motoro* Venom Apparatus

**DOI:** 10.3390/toxins10120544

**Published:** 2018-12-18

**Authors:** Filipe Silva, Yu Huang, Vítor Yang, Xidong Mu, Qiong Shi, Agostinho Antunes

**Affiliations:** 1 CIIMAR/CIMAR, Interdisciplinary Centre of Marine and Environmental Research, University of Porto, Av. General Norton de Matos, s/n, 4450-208 Porto, Portugal; filipecgsilva@gmail.com (F.S.); up201306198@fc.up.pt (V.Y.); 2 Department of Biology, Faculty of Sciences, University of Porto, Rua do Campo Alegre, s/n, 4169-007 Porto, Portugal; 3 Shenzhen Key Lab of Marine Genomics, Guangdong Provincial Key Lab of Molecular Breeding in Marine Economic Animals, BGI Academy of Marine Sciences, Shenzhen 518083, China; huangyu@genomics.cn (Y.H.); shiqiong@genomics.cn (Q.S.); 4 BGI Education Center, University of Chinese Academy of Sciences, Shenzhen 518083, China; 5 Pearl River Fisheries Research Institute, Chinese Academy of Fishery Sciences, Key Laboratory of Recreational Fisheries, Ministry of Agriculture, Guangdong Engineering Technology Research Center for Advanced Recreational Fisheries, Guangzhou 510380, China; muxd@prfri.ac.cn

**Keywords:** venom, transcriptomics, stingray, hyaluronidase, next-generation sequencing, selective pressure

## Abstract

Venomous animals are found through a wide taxonomic range including cartilaginous fish such as the freshwater stingray *Potamotrygon motoro* occurring in South America, which can injure people and cause venom-related symptoms. Ensuring the efficacy of drug development to treat stingray injuries can be assisted by the knowledge of the venom composition. Here we performed a detailed transcriptomic characterization of the venom gland of the South American freshwater stingray *Potamotrygon motoro*. The transcripts retrieved showed 418 hits to venom components (comparably to 426 and 396 hits in other two *Potamotrygon* species), with high expression levels of hyaluronidase, cystatin and calglandulin along with hits uniquely found in *P. motoro* such as DELTA-alicitoxin-Pse1b, Augerpeptide hhe53 and PI-actitoxin-Aeq3a. We also identified undescribed molecules with extremely high expression values with sequence similarity to the SE-cephalotoxin and Rapunzel genes. Comparative analyses showed that despite being closely related, there may be significant variation among the venoms of freshwater stingrays, highlighting the importance of considering elicit care in handling different envenomation cases. Since hyaluronidase represents a major component of fish venom, we have performed phylogenetic and selective pressure analyses of this gene/protein across all fish with the available information. Results indicated an independent recruitment of the hyaluronidase into the stingray venom relative to that of venomous bony fish. The hyaluronidase residues were found to be mostly under negative selection, but 18 sites showed evidence of diversifying positive selection (*P* < 0.05). Our data provides new insight into stingray venom variation, composition, and selective pressure in hyaluronidase.

## 1. Introduction

*Potamotrygon motoro*, commonly known as the ocellate river stingray, is a freshwater stingray with a circular disk, eyes raised from the dorsal surface, typically beige or brown, with dark-ringed orange spots [1]. It belongs to the Potamotrygonidae family that represents the only group of cartilaginous fish living mostly in freshwater. Freshwater stingrays occur in South America, within some river systems, each with its own endemic stingrays. Recent classifications of this family included five genera, *Heliotrygon*, *Paratrygon*, *Plesiotrygon*, *Potamotrygon* and *Styracura* [2,3]. One of the first described freshwater stingray was *P. motoro*, which is the most widespread species of the whole family across South America, found in freshwater rivers in Uruguay, Paraná-Paraguay, Orinoco, and Amazon River basins [1,4,5].

Similar to other species of the genus, the *P. motoro* possesses a tail with a sting comprehending the venom apparatus consisting of a rigid structure of dentin, serrated barbs, and an enveloping layer of cells with venom-producing capabilities [6,7]. Stingray injuries usually have two components, an immediate physical trauma from the penetration of the spine and an envenomation in the created wound when the spine tears through the integumentary tissue.

The stings will be used by the animal through tail-flicking towards the desired target if frightened, such as when there are capture attempts or close proximity of bathers. Unsuspecting individuals stepping on hidden spines in the sand is another possibility. The result is tissue breakdown and necrosis, followed by heavy pain and potentially further infection at the wound site [7,8,9]. The physical trauma is described as incredibly painful and can provoke severe damage due to the serrations of the stingers, which may strike vessels or nerves [7,10]. Furthermore, mucus surrounding the stingers and bacteria present in the epithelium can drastically worsen the condition of the wound. Bacterial infections are reported to commonly be gram-negative species with wide antibiotic resistance [11,12]. In areas without appropriate treatment or facilities available, or when no treatment is procured, the injuries’ severity increases, and death may occur. Edema, erythema, inflammation, vomiting, and headaches are other commonly described symptoms. Tetanus is also a reported risk [7,13,14,15,16].

Wounds related to freshwater stingrays are common in some South American regions in which these species are abundant, and the locals openly interact with them, such as in bathing areas or within fishing communities. Mishandling captive stingrays can also result in envenomation [8,9]. Actual confirmation of the species involved is difficult, but it is likely that many recorded occurrences are due to the highly widespread *Potamotrygon motoro* [15].

Despite the recent advances in venom studies, most species that are suspected of being venomous or have shown such potential remain largely unexplored. In particular, when it comes to aquatic species, from invertebrates such as coleoids to vertebrates such as scorpionfish, the number of analyzed species compared to the prospective amount is considerably reduced [17,18,19,20,21,22]. Moreover, in snake’s species venom composition could vary depending on where the sting occurred (different environments) and whether it was captive or wild specimens being handled, which could lead to a bigger array of compounds and symptoms [23,24]. Within stingrays, there have been studies isolating some of the molecules involved in their venoms and assessing their potential impact [25,26,27]. More recently, three transcriptomes were generated from the freshwater stingrays *P. amandae*, *P. falkneri* [28] and the marine blue-spotted stingray *Neotrygon kuhlii* [29], illuminating the molecular diversity of venoms in stingrays relatively to other venomous species.

Here, we present the venom gland transcriptome characterization of the South American freshwater stingray *P. motoro* revealing its venom components and performing comparative analyses to identify the main differences across members of the same genus (i.e., *Potamotrygon*). We assessed the protein coding transcript variability in the venom of the closely related *Potamotrygon* species to elucidate the venom composition of the *P. motoro* stingray that can easily injure humans, providing information required for the development of safety and treatment measures. Findings show slight variations that should be taken into consideration when approaching freshwater stingray envenomation cases and components that require further proteomic clarification.

We also analyzed the hyaluronidase (HYAL) gene family among fish due to its presence in the *P. motoro* venom gland and its relevance in the venom of other animals. The hyaluronidase enzyme is capable of hydrolyzing hyaluronan, an extracellular matrix component, lowering its viscosity [30]. Our results support an independent venom recruitment of the hyaluronidase in cartilaginous freshwater stingrays relatively to other fish. We identified the HYAL1, HYAL2 and HYAL6 paralogs, the last being a candidate for the gene recruitment into the venom molecular network. Furthermore, hyaluronidase key sites were detected as being under diversifying selective pressure. We hypothesized that such residues likely do not impact the hyaluronidase enzymatic function but could instead affect the protein stability, affinity, and protein-protein interaction.

## 2. Results and Discussion

### 2.1. Denovo Assembly

The Illumina HiSeq sequencing resulted in 38,674,474 paired-end reads of 90 bp each. After filtering out the adapters and low-quality sequences, a total of 35,977,224 clean reads were obtained for further processing. De novo assembly using Trinity produced 140,078 contigs with a length average of ~498 bp, minimum of 174 bp and maximum of 15,040 bp. There were 14543 contigs longer than 1000 bp. Of the contigs with expression higher than one fragment per kilobase of transcript per million mapped reads (FPKM), 27,032 potentially coding transcripts with an average length of ~787 bp were identified by the Trinotate annotation pipeline (Table 1).

Our sequencing and assembly data provided a comparable number of contigs to previous studies [28], with both significant expression and predicted coding regions. However, the average length of contigs and length of a large amount of our dataset was considerably lower, which could have resulted from the sampling and sequencing methods procedures considered.

### 2.2. Transcriptome Functional and Pathway Annotation

For the full annotation, the filtered contigs were subjected to BLAST [31] runs against the SwissProt [32], ToxProt [33] and the NCBI non-redundant protein databases [34]. Overall, 84.77% of the contigs were annotated against NCBI, 80.26% to SwissProt, 1.69% to ToxProt and 13.40% were left unannotated (Table 2). The top five species hits in the NCBI database were *Callorhinchus milii*, *Latimeria chalumnae*, *Lepisosteus oculatus*, *Xenopus tropicalis* and *Chrysemys picta bellii*, matching previous data with hits to fish (even though very few fish species were represented in the databases).

Similar to previous studies, little sequence diversity and closeness was found in the BLAST hits to the venom gland contigs (Figure S1) [28]. This is due to the lack of fish sequences in the SwissProt and the NCBI databases, which increases the complexity in the proper identification and characterization of venom components in species not closely related. Our data will assist identification in future studies but more effort in a higher diversity of species would be welcome.

### 2.3. Gene Ontology and Metabolic Pathways

**Figure 1:**
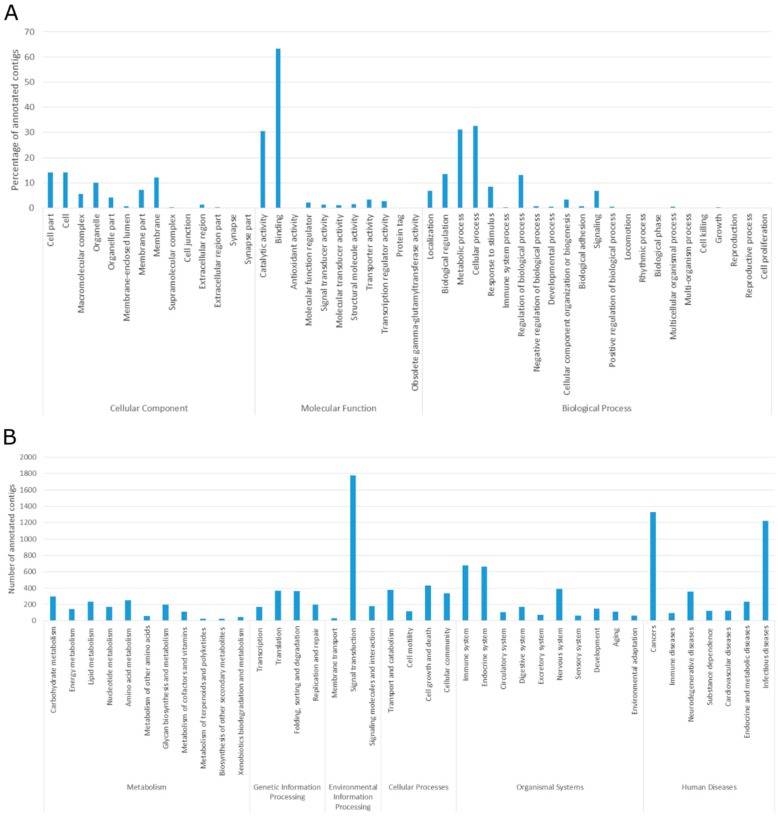
(**A**) Gene Ontology distribution of the coding region containing contigs retrieved from the freshwater stingray *Potamotrygon motoro* venom gland transcriptome. (**B**) Kyoto Encyclopedia of Genes and Genomes (KEGG) Classification of the coding region containing contigs of the freshwater stingray *Potamotrygon motoro* venom gland transcriptome.

Gene Ontology (GO) [35] analysis was performed to understand the functional properties of the contigs. Of the 27,032 predicted coding transcripts, 19,194 (77.60%) were successfully mapped into the three major functional groups: cellular component, molecular function, and biological process (Figure 1A).

**Figure 2:**
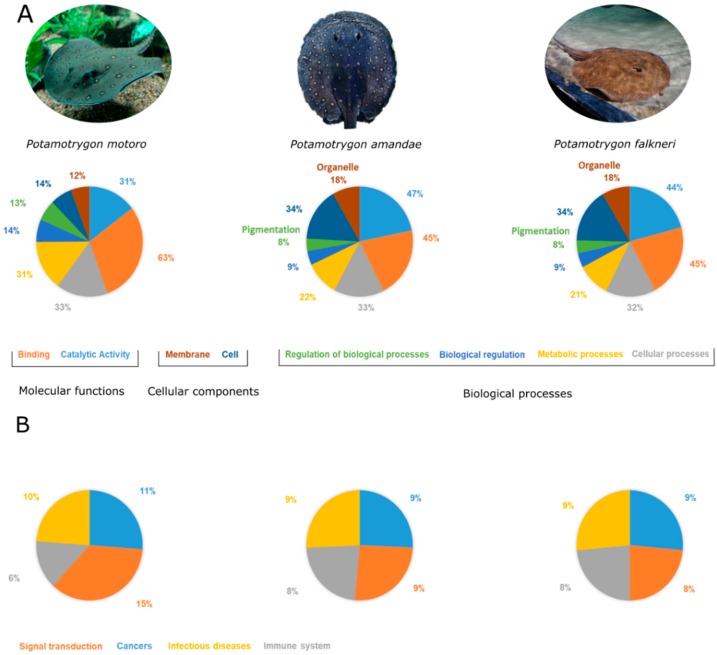
Graphical representation of the comparison between the most represented (**A**) gene ontology categories and (**B**) KEGG pathways found in *Potamotrygon motoro*, *Potamotrygon amandae* and *Potamotrygon falkneri* (data from the last two species reproduced from [26] 2009, Peptides). The percentage values correspond to the annotation values in each work for GO term and KEGG pathway annotation. The images of *P. motoro* (by Steven G. Johnson) and *P. falkneri* (by Andrew Kuchling) are used under the Creative Commons Attribution-Share Alike 3.0 Unported license. The image of *P. amandae* was reproduced from [1] 2013, Neotropical Ichtyhology.

GO terms annotation in the cellular components presented peaks for cell and membrane, with organelles closely following, a slight difference in comparison to *P. amandae* and *P. falkneri* (Figure 2A). As with the other members of the genus, cellular, metabolic, and biological regulation processes were the highest matching categories in biological processes. Regarding molecular functions, binding and catalytic activity were also the most mapped (Figure 1A).

The consistency found in the results of the transcriptomic characterization of three different stingray species of the *Potamotrygon* genus suggest that despite the inter-specific variation, key processes and components appear overabundant in the venom apparatus. 

The Kyoto Encyclopedia of Genes and Genomes (KEGG) [36] annotation can assist in the understanding of high-level functions and utilities of biological systems. Using the previous annotation retrieved from the SwissProt database it was possible to identify the KEGG pathways in which the transcripts were involved. There were 17,572 (71.04%) transcripts associated with KEGG pathways. Compared to the results obtained in [26], Cancers, Signal transduction, Immune system and Infectious diseases were still the most represented groups. However, the Endocrine system also reached values comparable to that of the Immune system (Figure 1B). It should also be noted that only around 6% of contigs were assigned to this pathway, compared to the 8% found in *P. amandae* and *P. falkneri* transcripts (Figure 2B). The distribution of the KEGG pathway annotation through the six major categories—Metabolism, Genetic Information Processing, Environmental Information Processing, Cellular Processes, Organismal Systems, and Human Diseases—differs from the other members of the genus, overall. Pathways involved in cancer were also the pathways with the highest matches, followed by the PI3K-Akt signaling pathway, HTLV-I infection, and Endocytosis. This suggests that while being closely related, the *Potamotrygon* genus possess significant diversity within its species and their venoms.

In respect to its metabolism, an overview of the KEGG pathway distribution showed 46 hits to the biosynthesis of amino acids and 138 to the biosynthesis of antibiotics. The amino acid metabolism is essential for the production of the venomous cocktail, while the antibiotic pathways is related with the production of bioactive components. There were also pathways identified in the carbon (78) and fatty acid (30) metabolisms.

The functional annotation of our dataset yielded a greater number of annotated contigs in KEGG pathways (17,572–71.0%) and in gene ontology (19,194–77.6%) compared to former works (KEGG: 14,131–56.3% and 13,147–59.5%; GO: 16,921–64.9% and 15,394–69.7%; *P. amandae* and *P. falkneri*, respectively) in the other two species of stingrays. This may be the underlying cause for the KEGG pathway and gene ontology to differ in relative values.

### 2.4. Venom Expression

The contigs of the *P. motoro* transcriptome were also searched for similarity to the UniProt ToxProt database, a curated list of toxins and venoms. High similarity hits included zinc metalloproteinases, hyaluronidases, venom prothrombin activator and alpha-latrocrustotoxin-Lt1a. These molecules play key roles in venom functionality. Several of the identified venoms have been previously described in stingrays. These include proteins involved in ion channels disruption and inhibition of muscle contraction, neuromuscular transmission and nervous system interference and affecting the circulatory system and its related processes [26]. The most abundant transcripts with hits to the database presented some common molecules with other species of the genus, such as cysteine-rich venom protein, cystatin-2, calglandulins, metalloproteinases, peroxiredoxin-4 and translationally controlled tumor protein homolog (Table 3). Interestingly, hyaluronidase is the second most abundant, but not the first as in the other two stingray species. Two particular transcripts that matched SE-cephalotoxin are highly expressed (Table 3). The fact that this molecule did not appear in the venom gland transcriptomes of both *P. amandae* and *P. falkneri* and possessed such high values led to a more in-depth identification of the coding region. BLAST searches with the sequence matched mostly hypothetical undescribed proteins, SE-cephalotoxin-like molecules and proteins encoded by the Rapunzel gene.

The reason the Rapunzel gene would be overrepresented in a venom gland transcriptome is not fully known. It is difficult to understand the underlying implications of this molecule primarily since it is not clearly identified through current databases. Assuming it is related to SE-cephalotoxin, then this result shows a very significant difference in venom composition within the *Potamotrygon* genus. It also represents a potential key component of the *P. motoro* venom. If similar to Rapunzel, a gene involved in bone structure formation, it will be necessary to determine first the function of the gene in stingrays and its implications as part of a venom cocktail. In the possibility of being another molecule, further studies would be required to understand its function and the possible evolutionary relationship with SE-cephalotoxin and Rapunzel. It should be noted that the hits to the NCBI database regarding SE-cephalotoxin-like genes were to non-venomous species and both these hits and those to Rapunzel, despite the low e-values (~1 × 10^−70^), showed only moderate sequence similarity (~40%).

Notably, we have not detected the presence of contigs in *P. motoro* with high similarities to phospholipase A2, previously described in other members of the genus, as well as in the common stingray *Dasyatis pastinaca*, and in envenomation scenarios causing symptoms such as hemorrhage and necrosis [37].

#### Unique Toxins Identified in *P. motoro* and Fish Venom Comparison

In addition to the SE-cephalotoxin/ Rapunzel transcripts, unexpected finds at high levels of expression included the DELTA-alicitoxin-Pse1b, Augerpeptide hhe53 and PI-actitoxin-Aeq3a. The transcripts for these molecules or similar have yet to be described in stingray venom. DELTA-alicitoxin-Pse1b is a pore-forming protein that can provoke hemolysis, making it a toxin focused on damaging the circulatory system [38]. Augerpeptide hhe53 is a protein expressed in venom ducts of the sea snail *Hastula hectica* and little is known about its function [39]. PI-actitoxin-Aeq3a is both a serine protease trypsin and potassium channel inhibitor and was originally described as a Kunitz-type serine protease inhibitor in the sea anemone *Actinia equine* [40], with consequences similar to those of the serine protease inhibitors, capable of affecting coagulation and the kinin system processes. Recently, *P. amandae* has been described as a distinct species from *P. motoro* that is believed to be a species complex [1]. Our results would sustain that hypothesis but could also be indicative of intraspecific variation.

It is worth mentioning that transcripts with similarity to venom phosphodiesterase were found, although at much lower expression values (<3.6 FPKM). Phosphodiesterase activity was previously found in the stingray *Urolophus halleri* [14] and in the bony fish *Scatophagus argus*, *Gymnapistes marmoratus* and *Synanceia horrida* [41]. However, there was no mention of these molecules in the most recent transcriptomic studies in stingrays, including those of the *Potamotrygon* genus. This protein is an enzyme with nuclease, pyrophosphatase and phosphatase activity and will hydrolyze nucleoside 5′-triphosphates and 5′-diphosphates, though not 5′-monophosphates. Together with 5′-nucleotidase, it has been reported as responsible for potential tissue necrosis and capable of inhibiting platelet aggregation induced by ADP [14,42,43,44].

Overall, within the high expression values, not including hyaluronidase, only phospholipase A2 of the key venom components found in bony fish (Table 4) was detected in the *Potamotrygon* species, although entirely absent from the *Potamotrygon motoro* transcriptome. Compared to the proteins of the marine stingray *Neotrygon kuhlii* extracted from the barb venom gland, only the cystatin and peroxiredoxin were found in common (Table 4).

The hyaluronidase transcripts showed elevated expression levels and this protein is believed to be a key component in the *Potamotrygon* genus venom. Thus, to better understand the hyaluronidase gene family evolution we performed phylogenetic analyses across all fish for which the hyaluronidase gene information is publicly available.

### 2.5. Hyaluronidase Phylogeny and Selective Pressure

**Figure 3:**
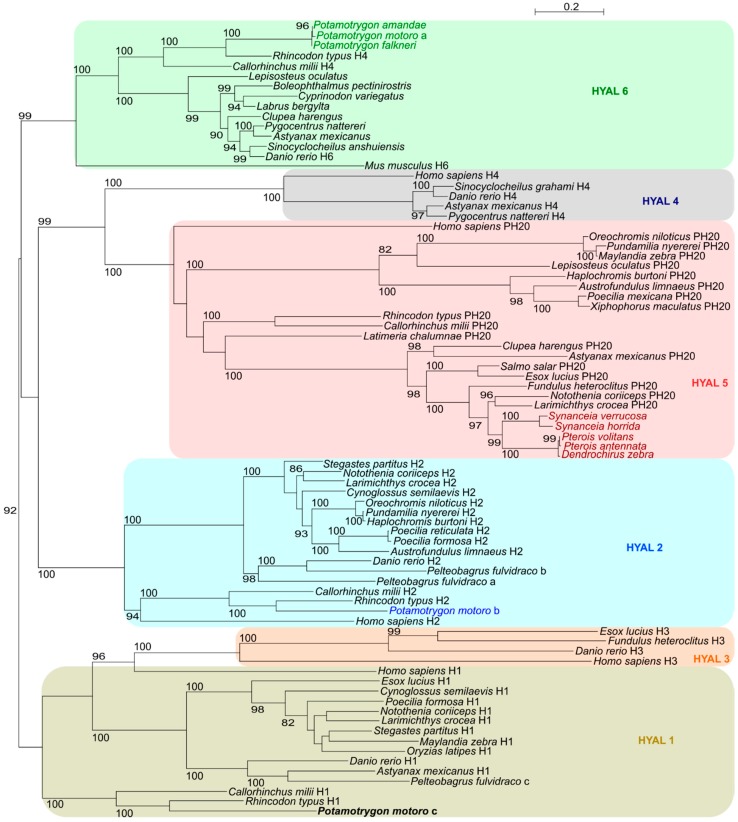
Maximum likelihood tree reconstruction of the hyaluronidase protein family (80 amino acid sequences of 529 residues) with 1000 bootstrap replications. Only branch supports higher than 80 are shown. Identified enzyme functional groups are labelled after each species. Highly expressed hyaluronidase sequences are indicated by green colored names. Known venomous fish hyaluronidase sequences are indicated by red colored names. *P. motoro* sequence with similarity to HYAL2 is highlighted with a blue colored name. HYAL1 sequence from *P. motoro* is indicated in bold. In the clade containing HYAL6, given the clade resolution and the phylogenetic proximity of *Danio rerio* H6 and *Mus musculus* H6, the HYAL4 designation for the *Rhincodon typus* and the *Callorhinchus milii* are considered mislabels.

We estimated a phylogenetic reconstruction of a total of 80 hyaluronidases sequences found across fish and mammal representatives of all HYAL functional groups using maximum likelihood methods. The produced trees strengthened the proximity previously inferred among hyaluronidase present in the venomous glands of Scorpaeniformes and the PH-20 isotype (HYAL5) [46], located on the sperm surface acting as a receptor for the zona pellucida surrounding the oocyte and responsible for the degradation of extracellular matrix (ECM) during oocyte fertilization [47]. It also reinforced the idea that the hyaluronidases found in the venoms of stingrays are not immediately related to the hyaluronidases of venomous bony fish, in particular those found in Scorpaeniformes. The ocellate river stingray *P. motoro* possessed three well-defined sequences of hyaluronidase. Interestingly, the phylogenetic analyses placed the most expressed transcript together with a zebrafish HYAL6 sequence in a clade, with no proximity to any potentially venomous fish (Figure 3). Another transcript was grouped in the clade containing the HYAL2 sequences and together with its low expression level indicates a likelihood of this particular protein being involved in house-keeping activities. Similarly, the other transcript was likely also part of house-keeping processes. We were unable to properly resolve the hyaluronidase group for this sequence. This may be due to the distance between cartilaginous and bony fish and the similarity of the HYAL1 and HYAL3 groups. BLAST identification indicated the transcript to be similar to the HYAL1 group sequences. These results do not create a monophyletic group with other venomous animals, supporting previous transcriptomic studies in stingrays [28,29] and the more precise phylogenetic study of venomous fish that showed up to 18 independent origins (convergent evolution) of venom in fish [48].

Interestingly, the fact that the most expressed type of hyaluronidase (HYAL6) found in the venom gland transcriptome of all three freshwater stingrays were all grouped together suggests a common origin of the venom specialization. The amino acid sequence similarity and the phylogenetic proximity to the zebrafish HYAL6 sequence compared to the other types of HYAL suggests this gene as a candidate for the hyaluronidase recruitment into the venom of the *Potamotrygon* lineage. However, data in fish regarding hyaluronidase is limited, especially in HYAL6. The sequences besides the zebrafish found in the group were mostly predicted hyaluronidase-like and with no group indicated. Given the support for both *Danio rerio* H6 and *Mus musculus* H6 phylogenetic position and the resolution of the hyaluronidase family members, it is likely that this group represents HYAL6. The *Rhincodon typus* and the *Callorhinchus milii* sequences were likely incorrectly annotated, potentially due to the absence of HYAL6 in the annotation database at the time, as it is a pseudogene in *Homo sapiens* (i.e., no protein sequence available). Performing a BLAST with *Mus musculus* sequence against the NCBI database returns only hits to itself and a HYAL6 sequence from *Rattus norvegicus*, while the remaining hits represent HYAL4, suggesting high sequence similarity and the likely reason for the annotation found in the cartilaginous fish. Regardless, this is the first identification of a potentially recruited gene that would also match with an independent evolution of venom in fish. The hyaluronidases of venomous bony fish were found to be closely related to the HYAL5 as opposed to HYAL6 (Figure 3), indicating that different groups in the hyaluronidase family were recruited into venoms. Currently, little is known about HYAL6 other than it is highly expressed in murine testicular tissue together with HYAL5. HYAL6 was determined to not have hyaluronidase activity when pH values were neutral [49].

Using the HyPhy software package [50], several selection analyses algorithms were used. As expected with coding sequences, most sites were found to be under negative selection ensuring that a functional molecule is encoded. Results from Single Likelihood Ancestor Counting (SLAC), Random Effects Likelihood (REL), Fixed Effects Likelihood (FEL) [51] and Fast Unconstrained Bayesian AppRoximation (FUBAR) [52] analyses did not reveal any sites with evidence of positive selection (317, 529, 374 and 415 negatively selected sites, respectively). The prominent negative selection profile may have masked possible sites under positive selection and thus the Mixed Effects Model of Evolution (MEME) [53] model was used to identify sites under episodic diversifying selection (codons: 20, 53, 58, 79, 103, 113, 114, 120, 159, 179, 190, 234, 245, 488, 501, 505, 523 and 526).

**Figure 4:**
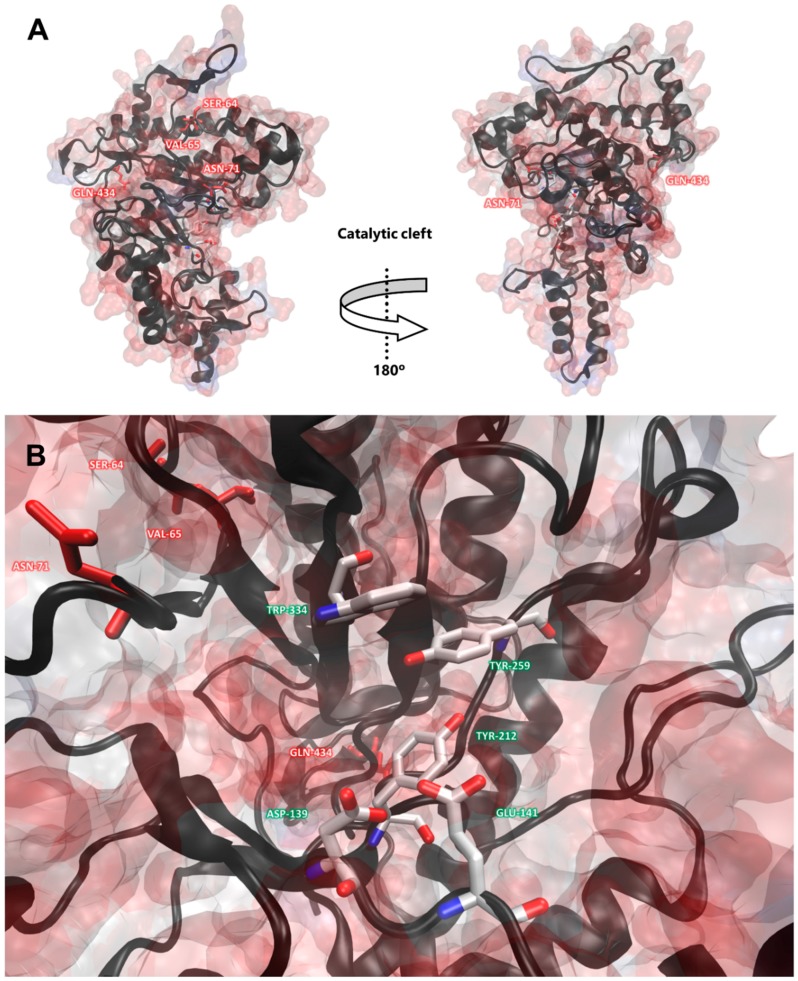
The three-dimensional protein model of the *Potamotrygon motoro* hyaluronidase inferred by Phyre2 [55]. Positively selected amino acids (*P *< 0.01) are colored in red and the catalytic residues are represented in white (green label). Images were generated using the Visual Molecular Dynamics software [56]. (**A**) Side-view of the catalytic cleft and protein overview. (**B**) Catalytic cleft close look.

The three-dimensional protein structural modelling of hyaluronidase allowed the visualization of the potential location of the specific residues. To further improve the accuracy of the residue selective pressure, only sites still significant with *P* < 0.01 were marked (113, 114, 120 and 505; 523 could not be modelled). The residues under diversifying selection were found mostly in the protein surface. Interestingly, some of the residues under positive selection were found to be close to each other in the modelled three-dimensional structure (Figure 4). This pattern corroborates a relation between positive selection and the protein secondary structure elements studied in *Drosophila*. It was found that amino acids in disordered regions had a higher chance to be under positive selection in relation to their proportion in the proteins. In comparison, residues in helices and beta-structures had less sites under positive selection than expected. It is noteworthy to mention that sites under diversifying pressure occurred close to each other in the protein sequence more often than anticipated [54].

Inspecting the protein model revealed that the positively selected sites were in the proximity of the catalytic region. Although the proximity to the catalytic region could mean these sites affect the protein activity, it does not directly translate to a guaranteed effect. However, changes to these residues may result in slight structural shifts, altering interactions with other molecules, the molecule stability or affecting the orientation of the catalytic residues. Our results suggest that the enzymatic reaction is conserved, and the modifications required for the protein venom role would be optimizations on the surface recognition and related protein-protein interactions.

The fact that these changes are observed across an analysis of multiple family members of hyaluronidase involved in different processes, but all catalyzing the degradation of hyaluronic acid, shows that altering the tissue permeability is a key factor in a wide variety of processes, ranging from usual metabolic pathways to venomous activity.

This demonstrates that while hyaluronidase recruitment into venoms in fish was likely independent from other venomous animals, not all genes part of the venom arsenal had to undergo rapid mutation to retain relevance. For example, the king cobra genome showed that the hyaluronidase was not found under diversifying selection, suggesting that auxiliary genes not causing resistance in the targets are under lower selective pressure [57]. This would also be insightful to understand the multiple cases of convergent evolution seen in venoms across a wide taxonomic array.

Hyaluronidase has been recruited into venoms along a wide range of taxonomic groups and it has been suggested to use the protein as a therapeutic target [58]. This convergent evolution suggests that the molecule is well suited for an important role in venoms, whether as a facilitating agent or something else. The high expression values found in the venom glands transcriptomes of the freshwater stingrays and the small number of positively selected residues, along with their location in the protein structure, strongly suggests that hyaluronidase plays a more important role than previously recognized, which should be further assessed in future proteomic studies.

## 3. Conclusions

The transcriptomic characterization of the *P. motoro* venom gland revealed that despite the species being closely related to other freshwater stingrays (i.e., same genus) there might be significant variation in the venom composition among species. Notably, we found contigs with hits to sequences of DELTA-alicitoxin-Pse1b, Augerpeptide hhe53, PI-actitoxin-Aeq3a and SE-cephalotoxin with significant expression levels, when compared to the most expressed molecules of *P. amandae* and *P. falkneri*. The most notable absence in the venom of *P. motoro* was the phospholipase A2. The venom gland of *P. motoro* possessed multiple transcripts that were mapped to the pathways of antibiotic synthesis, such as terpenoid and polyketide metabolism, and biosynthesis of secondary metabolites, in particular the pathways of penicillin, cephalosporin, streptomycin, neomycin, kanamycin, and gentamicin. Also identified were pathways related to diseases, these being cancers, immune diseases, substance dependence, cardiovascular diseases, endocrine and metabolic diseases, neurodegenerative and infectious diseases.

Detailed analyses of the hyaluronidase showed that its recruitment into the venom of freshwater stingrays was an independent event relatively to other venomous bony fish species. The hyaluronidase genes have been the target of diversifying selection, indication that across duplications and gene mutations, these molecules were recruited and adjusted into several different processes. Interestingly, there is no evidence suggesting that the protein optimization required changes to the original enzymatic reaction. Rather, the molecule structural stability, substrate affinity and protein-protein interaction would be the elements most likely to have changed.

Our work expands the existing knowledge in cartilaginous fish venom composition. It also reinforced the importance of hyaluronidase in venoms, evidence of its independent recruitment and indication that not all venom components are subject to high diversifying pressures. The knowledge of venom composition of fish is still very narrow given the huge diversity of venomous species. In particular, to fully understand all the independent specializations occurring in this group more information on the involved molecules and their sequences is required. The more data becomes available the clearer the role and potential of the bioactive components used by venomous animals, such as freshwater stingrays.

## 4. Materials and Methods

### 4.1. Fish Collection and Sample Processing

Here we studied the venomous freshwater stingray *P. motoro* (PM, Taxonomy ID: 86373). The fish was bred by the Pearl River Basin Sub-center, National Sharing Service Infrastructure of Fishery Germplasm Resources, Guangzhou, China. 

The species identification was supported by both morphology and DNA barcoding experiments (with identity > 99%). Overall, 10 spine samples, including the venom glands (one from each fish), were dissected, immediately snap-frozen and deposited in liquid nitrogen tanks for future processing. All the collection and processing procedures have been reviewed and approved by the Institutional Review Board on Bioethics and Biosafety of BGI (No. BGI-IRB 15139; 27 November 2015).

### 4.2. RNA Extraction and Sequencing

Total RNA was isolated from the 10 pooled venom glands using TRIzol^®^ LS Reagent (Invitrogen, Carlsbad, CA, USA) and the quality of each RNA sample was assessed by Agilent 2100 Bioanalyzer (Agilent Technologies, Palo Alto, CA, USA). Afterwards, purification and isolation of mRNA with poly(A) tails was performed using Oligo-(dT)-attached magnetic beads, and the obtained mRNA was proceeded to Illumina cDNA library construction and sequencing through Illumina HiSeq2000 platform (Illumina, San Diego, CA, USA) at BGI-Tech (BGI, Shenzhen, Guangdong, China). The lack of a well-defined gland structure makes tissue extraction challenging and thus not all the molecules found may be representative of the stingray venom [59,60].

### 4.3. Data Filtering and De novo Assembly

Paired-end raw reads (China National Genebank Database, project accession CNP0000235) generated from the sequencing platform were filtered with SOAPnuke [61] to remove the junk reads with adaptors, more than 10% of N bases and more than 50% of low-quality bases (base quality score ≤ 10). The remaining clean data were then de novo assembled into contigs using the Trinity software (v2.1.1) with the specific parameter set to “--min_contig_length 150” and others set to default [35]. TGICL software (v2.0) was used to eliminate the redundancies in the assembly with given parameter “-v 25 -O ‘-repeat_stringency 0.95’” [62].

### 4.4. Transcript Expression Calculation

Expression values were calculated by mapping the raw reads against the assembled contigs using CLC’s Genomics Workbench (v9.5.2) RNA-Seq analysis option. Multiple statistics were obtained, including fragments per kilobase of transcript per million mapped reads.

### 4.5. Transcriptome, Functional and Pathway Annotations

The transcripts were subject to a filter dependent on their expression, in which only contigs with values higher than 1 FPKM were considered for further analyses. The remaining contigs were then processed through Trinity’s Trinotate software [63] to fully annotate and to determine the function and pathways of the molecules encoded by the transcripts retrieved from the venom gland. The annotation part of this process included the necessary SwissProt [32] and Pfam [64] databases, as well as NCBI’s non-redundant protein database [34] (restricted to vertebrates) and Uniprot’s ToxProt [33]. All of these were target databases for the contigs’ BLAST analysis [31]. Through this process, the TransDecoder [65] software determined coding regions in the contigs, retaining only opening reading frames (ORFs) longer than 100 amino acids. GO [35] and pathway data (KEGG) [36] was extracted from hits to the SwissProt and Pfam databases. GO term annotation results were exported using WEGO webserver [66].

### 4.6. Sequence Alignment and Phylogenetic Analyses

Nucleotide sequences corresponding to hyaluronidases were retrieved from the GenBank [34] and RefSeq [67]. The search was performed using BLAST. The search queries were the coding sequences (CDS) of hyaluronidases from *Synanceia horrida* already available in GenBank and the hyaluronidase transcripts from *P. motoro*. Representative sequences from different hyaluronidase functional groups were manually searched for and retrieved. The *Homo sapiens* sequences were used to more accurately identify functional HYAL groups. The *Mus musculus* HYAL6 was used instead of the human HYAL6 that is a pseudogene. All sequences used, and their accession or reference, are found in Table S1.

Nucleotide sequences were translated into amino acid sequences and aligned using the CLUSTAL [68] algorithm within SEAVIEW [69] (v4.5 4), which was also used for visualization and manual editing of the sequences [70]. Regions in which homology could not be guaranteed were removed from further analyses.

The most appropriate model of protein evolution for Maximum Likelihood phylogenetic trees was chosen using ProtTest [71,72], which returned WAG + I + G. Maximum Likelihood phylogenetic trees were reconstructed using IQ-TREE [73]. Maximum likelihood tree branches were supported by 1000 bootstraps.

### 4.7. Selective Pressure Analyses

In the event of recombination, single tree topologies cannot explain the evolutionary path of the recombined sequence. In this scenario, the likelihood-ratio test (LRT) viability may be doubtful. LRT was shown to be robust at low levels of recombination (less than three recombination events with 10 sequences) [74]. To account for possible interference, a genetic algorithm for recombination detection (GARD) and Single Breakpoints (SBP) algorithms [75,76,77,78,79] of the HyPhy [50] multiplatform were used. When relevant levels of recombination were found, sequences were partitioned prior to the selection analyses. 

The HyPhy multiplatform was used to assess selective pressure, running the SLAC algorithm, a derivative of the Suzuki—Gojobori counting approach; the FEL algorithm, estimating non-synonymous and synonymous substitution rates at each site; the REL algorithm [51,78,79], which categorizes non-synonymous and synonymous rates variation across all sites and infers selective pressure in sites using an empirical Bayes approach; and the FUBAR algorithm [52,76,77,79], which uses a Markov chain Monte Carlo method and allows visualization of Bayesian inference for each site. To detect episodic diversifying selection, masked by heavy purifying pressure, the MEME algorithm [53,76,77,78,79] was also used.

### 4.8. Structure Modelling

Computational structural methods have been successfully used to identify relevant molecular interactions of protein involved in complex pathways such as toxin target and protein inhibition mechanisms [77,78,79,80,81]. The three-dimensional (3D) molecular structure of hyaluronidase was predicted using the Phyre2 webserver (normal modelling mode) [55,77,78,79]. The obtained models were viewed using VMD—Visual Molecular Dynamics [56]. Amino acids under positive selection were marked in the structure for posterior analysis. Hyaluronidase catalytic residues were annotated accordingly to The Catalytic Site Atlas 2.0 [82].

## Figures and Tables

**Table 1:** Statistics of the freshwater stingray *Potamotrygon motoro* venom gland transcriptome sequencing and assembly (retrieved from the Trinotate annotation pipeline).

Process	Value
Number of raw reads	38,674,474
Raw data (bp)	3,480,702,660
Read length (bp)	90
Number of high-quality reads	35,977,224
Average high-quality read length (bp)	80
Number of contigs	140,078
Number of contigs ≥ 1 FPKM	107,129
Number of contigs ≥ 1 FPKM and containing coding sequences	27,032
Contigs (bp)	69,861,618
N50	690
Average contig length (bp)	498
Min. contig length (bp)	174
Max. contig length (bp)	15,040

**Table 2:** Statistics of the coding region containing contigs annotated in the venom gland transcriptome of the freshwater stingray *Potamotrygon motoro*.

Database	Number of Annotated Contigs
NCBI	20,967 (84.77%)
SwissProt	19,851 (80.26%)
NCBI and SwissProt	19,398 (78.43%)
NCBI or SwissProt	21,420 (86.60%)
ToxProt	418 (1.69%)
PFAM	16,823 (68.02%)
GO	19,194 (77.60%)
KEGG	17,572 (71.04%)

**Table 3:** Top 25 most expressed transcripts from the *Potamotrygon motoro* venom gland transcriptome with hits to ToxProt. The corresponding Top 25 transcripts from *P. amandae* and *P. falkneri* are also shown (retrieved from [26]). Rows marked in red indicate differences in the most expressed molecules for each species.

*Potamotrygon motoro*				*Potamotrygon amandae*			*Potamotrygon falkneri*		
Transcript	Protein	Uniprot Accession	FPKM	Protein	Uniprot Accession	FPKM	Protein	Uniprot Accession	FPKM
TR1637	SE-cephalotoxin	B2DCR8	42139.08	Hyaluronidase	J3S820	22201.33	Hyaluronidase	J3S820	9488.18
TR45956	Hyaluronidase	J3S820	7271.53	Translationally controlled tumor protein homolog	U3EQ60	1225.86	Hemolytic toxin Avt-1	Q5R231	757.19
TR7202	SE-cephalotoxin	B2DCR8	489.97	Cysteine-rich venom protein latisemin	Q8JI38	891.77	Translationally controlled tumor protein homolog	U3EQ60	734.38
TR53378	DELTA-alicitoxin-Pse1b	P0DL56	399.42	Venom allergen 5	P81656	854.22	Calglandulin	Q3SB11	644.06
TR15069	Cystatin-2	J3SE80	373.92	Calglandulin	Q8AY75	835.98	Putative Kunitz-type serine protease inhibitor	B2BS84	450.57
TR10238	Augerpeptide hhe53	P0CI21	324.45	Cystatin-2	J3SE80	629.15	Peroxiredoxin-4	P0CV91	397.49
TR3474	Translationally controlled tumor protein homolog	U3EQ60	315.67	Hemolytic toxin Avt-1	Q5R231	401.11	Cysteine-rich venom protein 1	Q8T0W5	386.31
TR112682	Calglandulin	Q3SB11	202.31	Putative Kunitz-type serine protease inhibitor	B2BS84	354.6	Venom allergen 5	P81656	357.2
TR24275	Putative Kunitz-type serine protease inhibitor	B2BS84	198.6	Peroxiredoxin-4	P0CV91	304.79	Calglandulin	Q3SB11	318.87
TR20805	Venom allergen 5	P81656	174.48	Cysteine-rich venom protein 1	Q8T0W5	211.07	Cysteine-rich venom protein latisemin	Q8JI38	299.5
TR20804	Venom allergen 5.02	P35782	165.85	Alpha-latroinsectotoxin-Lt1a	Q02989	206.6	Vespryn	Q2XXL4	172.92
TR14068	PI-actitoxin-Aeq3a	P0DMW6	158.3	Kunitz-type serine protease inhibitor bitisilin-3	Q6T269	141.02	Venom serine protease 34	Q8MQS8	156.83
TR15070	Cystatin-2	J3SE80	146.47	Analgesic polypeptide HC3	C0HJF3	121.14	Calglandulin	Q8AY75	150.86
TR86455	Insulin-like growth factor-binding protein-related protein 1	G4V4G1	142.04	Kunitz-type serine protease inhibitor kappa-theraphotoxin-Hh1a	P68425	117.78	Alpha-latrocrustotoxin-Lt1a	Q9XZC0	150.43
TR110388	Calglandulin	Q3SB11	128.78	Venom prothrombin activator porpharin-D	Q58L93	104.65	Alpha-latrotoxin-Lh1a	G0LXV8	150.2
TR112679	Calglandulin	Q3SB11	126.9	Zinc metalloproteinase-disintegrin-like BmMP	A8QL49	101.89	Zinc metalloproteinase-disintegrin-like BmMP	A8QL49	143.04
TR113284	Cysteine-rich venom protein 1	Q8T0W5	82.72	Acidic phospholipase A2	Q9DF56	100.95	Cystatin-2	J3SE80	140.7
TR67254	Putative Kunitz-type serine protease inhibitor	B2BS84	73.96	Vespryn	Q2XXL4	95.12	Venom protease	Q7M4I3	118.09
TR9752	Zinc metalloproteinase-disintegrin-like BmMP	A8QL49	69.87	Insulin-like growth factor-binding protein-related protein 1	G4V4G1	93.55	Alpha-latroinsectotoxin-Lt1a	Q02989	114.49
TR1967	Alpha-latrocrustotoxin-Lt1a	Q9XZC0	68.33	Delta-latroinsectotoxin-Lt1a	Q25338	87.69	Kunitz-type serine protease inhibitor HNTX-852	P0DJ69	108.68
TR119403	Alpha-latrocrustotoxin-Lt1a	Q9XZC0	68.3	Ohanin	P83234	86.84	Venom prothrombin activator porpharin-D	Q58L93	107.52
TR110386	Calglandulin	Q3SB11	67.47	Venom prothrombin activator vestarin-D2	A6MFK8	71.33	Analgesic polypeptide HC3	C0HJF3	106.37
TR7292	DELTA-thalatoxin-Avl1a	Q5R231	66.92	Blarina toxin	Q76B45	70.84	Snake venom metalloprotease inhibitor	A8YPR9	86.64
TR53095	Peroxiredoxin-4	P0CV91	64.91	Venom protease	Q7M4I3	59.88	Kunitz-type serine protease inhibitor bitisilin-3	Q6T269	81.19

**Table 4:** Relevant protein venom components found in bony fish (22 species) and the marine stingray *Neotrygon kuhlii*. Molecules found in the freshwater stingrays (this study and [26]) are indicated in red.

Venom Components	
Bony Fish [41]	Marine Stingray [29]
Trachynilysin (TLY)	60S acidic ribosomal protein
Stonustoxin (SNTX)	ATP synthase
Verrucotoxin (VTX)	Coronin
Neoverrucotoxin (neoVTX)	Cystatin
Cardioleputin	Cytochrome C
Nocitoxin	Ferritin
Karatoxin	Galectin
Sp-CTx	Ganglioside GM2 activator
Plumieribetin	Glutathione S-transferase mu
SP-CL 1-5	Hemoglobin subunit alpha
Dracotoxin	Leukocyte elastase inhibitor
Trachinine	Nucleoside diphosphate kinase
SA-HT	Peroxiredoxin 6
TmC4-47.2	Transaldolase
Nattectin	Type III intermediate filament
Toxin-PC	Voltage-dependent anion channel
Wap65	-
Natterin	-
Hyaluronidase	-
Phospholipase A2 [45]	-
Proenkephalin [45]	-
Neuropeptide Y [45]	-

## Supplementary Materials

The following data are available online at http://www.mdpi.com/2072-6651/10/12/544/s1, Figure S1: Hits distribution representation per species following BLAST searches of the *Potamotrygon motoro* transcriptome against the SwissProt database; Table S1: Accession numbers for the sequences used in phylogenetic and selective pressure analyses.
