# Supplementary material for: Transcriptomic Characterization of the South American Freshwater Stingray Potamotrygon motoro Venom Apparatus
Source: Toxins (Basel). 2018 Dec 18;10(12):544. doi: 10.3390/toxins10120544 (PMC6315956; doi:10.3390/toxins10120544)
Supplement: Supplementary file 1 [file toxins-10-00544-s001.pdf]

# Supplementary Materials: Transcriptomic characterization of the South American freshwater stingray *Potamotrygon motoro* venom apparatus

**Filipe Silva, Yu Huang, Vítor Yang, Xidong Mu, Qiong Shi and Agostinho Antunes**

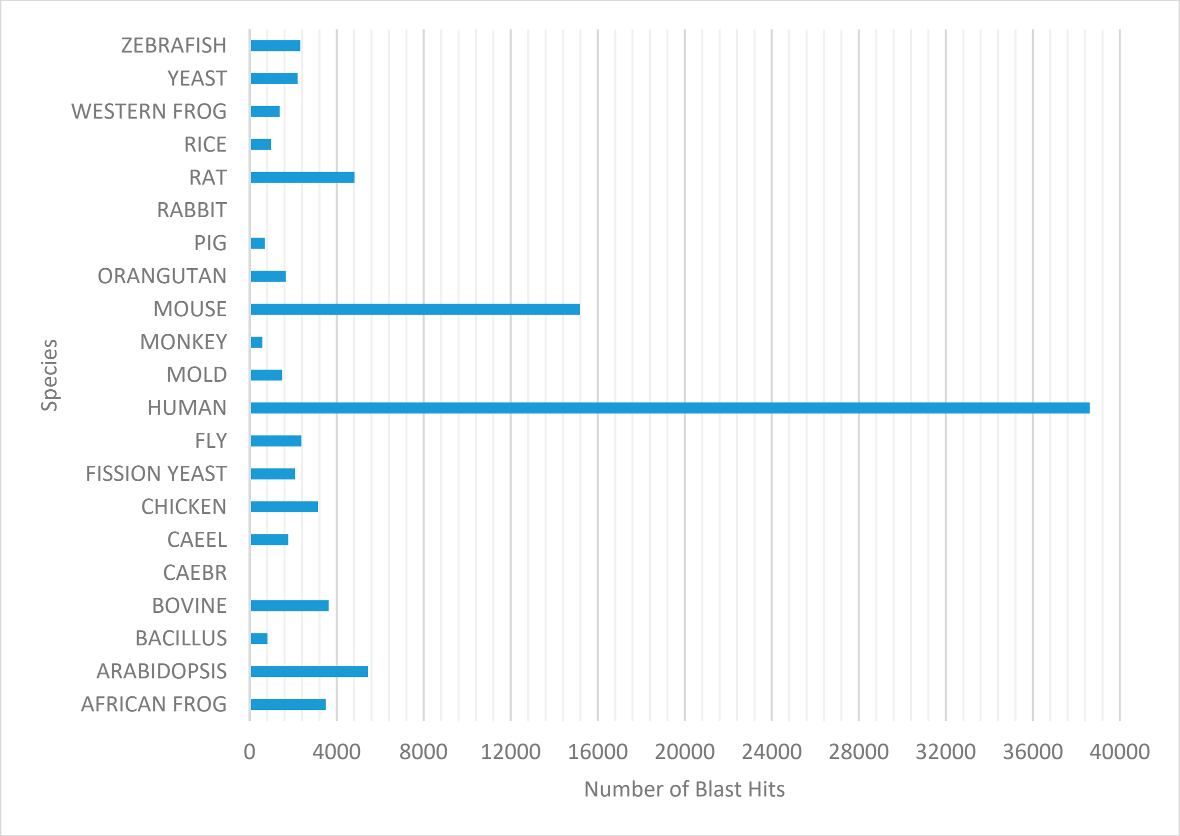


**Figure S1.** Hits distribution representation per species following BLAST searches of the Potamotrygon motoro transcriptome against the SwissProt database.

**Table S1.** National Center for Biotechnology Information accession numbers for the sequences used in phylogenetic and selective pressure analyses.

| Organism | Functional Group | Accession Number |
|---|---|---|
| *Potamotrygon motoro* | All | CNP0000235 (China National Genebank Database) |
| *Potamotrygon amandae* | H6 | SRR2039259 |
| *Potamotrygon falkneri* | H6 | SRR2039259 |
| *Pelteobagrus fulvidraco* | All | SRP057554 |
| *Dendrochirus zebra* | PH-20 | Available on request |
| *Pterois antennata* | PH-20 | AB759697.1 |
| *Pterois volitans* | PH-20 | AB759698.1 |
| *Synanceia horrida* | PH-20 | AY232496.1 |
| *Synanceia verrucosa* | PH-20 | AB607856.1 |
| *Oryzias latipes* | H1 | XM_011474790.1 |
| *Astyanax mexicanus* | H1 | XM_007234299.1 |

| | | |
|---|---|---|
| *Astyanax mexicanus* | H4 | XM_007246551.3 |
| *Astyanax mexicanus* | H6 | XM_022672245.1 |
| *Astyanax mexicanus* | PH-20 | XM_007246550.1 |
| *Clupea harengus* | PH-20 | XM_012822748.1 |
| *Clupea harengus* | H6 | XM_012822752.1 |
| *Latimeria chalumnae* | PH-20 | XM_006013119.2 |
| *Danio rerio* | H1 | NM_001039997.1 |
| *Danio rerio* | H2 | XM_682548.6 |
| *Danio rerio* | H3 | XM_009303978.1 |
| *Danio rerio* | H4 | XM_017354271.1 |
| *Danio rerio* | H6 | NM_001080671.1 |
| *Austrofundulus limnaeus* | H2 | XM_014006413.1 |
| *Austrofundulus limnaeus* | PH-20 | XM_014007943.1 |
| *Fundulus heteroclitus* | H3 | XM_012866741.1 |
| *Fundulus heteroclitus* | PH-20 | XM_012874928.1 |
| *Poecilia formosa* | H1 | XM_007550448.1 |
| *Poecilia formosa* | H2 | XM_007550436.1 |
| *Poecilia mexicana* | PH-20 | XM_014979450.1 |
| *Poecilia reticulata* | H2 | XM_008408657.1 |
| *Xiphophorus maculatus* | PH-20 | XM_005810480.1 |
| *Esox lucius* | H1 | XM_010896077.1 |
| *Esox lucius* | H3 | XM_010895839.2 |
| *Esox lucius* | PH-20 | XM_010864430.1 |
| *Lepisosteus oculatus* | PH-20 | XM_006633404.1 |
| *Lepisosteus oculatus* | H6 | XM_015352485.1 |
| *Haplochromis burtoni* | H2 | XM_005927429.1 |
| *Haplochromis burtoni* | PH-20 | XM_005933571.2 |
| *Larimichthys crocea* | H1 | XM_010745848.1 |
| *Larimichthys crocea* | H2 | XM_010745849.1 |
| *Larimichthys crocea* | PH-20 | XM_010742185.1 |
| *Maylandia zebra* | H1 | XM_004548653.3 |
| *Maylandia zebra* | PH-20 | XM_014409614.1 |
| *Notothenia coriiceps* | H1 | XM_010778234.1 |
| *Notothenia coriiceps* | H2 | XM_010778233.1 |
| *Notothenia coriiceps* | PH-20 | XM_010790710.1 |
| *Oreochromis niloticus* | H2 | XM_003448316.2 |
| *Oreochromis niloticus* | PH-20 | XM_013271188.1 |
| *Pundamilia nyererei* | H2 | XM_005724561.1 |
| *Pundamilia nyererei* | PH-20 | XM_013908758.1 |
| *Stegastes partitus* | H1 | XM_008289283.1 |
| *Stegastes partitus* | H2 | XM_008289310.1 |
| *Cynoglossus semilaevis* | H1 | XM_008319590.1 |
| *Cynoglossus semilaevis* | H2 | XM_008319591.1 |
| *Salmo salar* | PH-20 | XM_014169991.1 |
| *Labrus bergylta* | H6 | XM_020634952.1 |
| *Pygocentrus nattereri* | H6 | XM_017713623.1 |
| *Pygocentrus nattereri* | H4 | XM_017713657.1 |
| *Sinocyclocheilus anshuiensis* | H6 | XM_016499280.1 |
| *Cyprinodon variegatus* | H6 | XM_015386515.1 |
| *Boleophthalmus pectinirostris* | H6 | XM_020932069.1 |

| | | |
|---|---|---|
| *Sinocyclocheilus grahami* | H4 | XM_016290273.1 |
| *Homo sapiens* | H1 | AF118821.1 |
| *Homo sapiens* | H2 | BC000692.2 |
| *Homo sapiens* | H3 | NM_003549.3 |
| *Homo sapiens* | H4 | BC104788.1 |
| *Homo sapiens* | PH-20 | NM_153189.2 |
| *Mus musculus* | H6 | NM_028920.2 |
| *Rhincodon typus* | H1 | XM_020534579.1 |
| *Rhincodon typus* | H2 | XM_020525397.1 |
| *Rhincodon typus* | PH-20 | XM_020527772.1 |
| *Rhincodon typus* | H6 | XM_020527778.1 |
| *Callorhinchus milii* | H1 | XM_007890236.1 |
| *Callorhinchus milii* | H2 | XM_007890411.1 |
| *Callorhinchus milii* | PH-20 | XM_007898665.1 |
| *Callorhinchus milii* | H6 | XM_007898555.1 |
